# Fenofibrate Increases the Population of Non-Classical Monocytes in Asymptomatic Chagas Disease Patients and Modulates Inflammatory Cytokines in PBMC

**DOI:** 10.3389/fcimb.2021.785166

**Published:** 2022-03-11

**Authors:** Azul V. Pieralisi, Ágata C. Cevey, Federico N. Penas, Nilda Prado, Ana Mori, Mónica Gili, Gerardo A. Mirkin, Juan Gagliardi, Nora B. Goren

**Affiliations:** ^1^ Universidad de Buenos Aires. Facultad de Medicina. Departamento de Microbiología, Parasitología e Inmunología, Buenos Aires, Argentina; ^2^ CONICET Universidad de Buenos Aires. Instituto de Investigaciones Biomédicas en Retrovirus y SIDA (INBIRS), Buenos Aires, Argentina; ^3^ Division of Cardiology, Hospital del Gobierno de la Ciudad de Buenos Aires "Dr. Cosme Argerich", Buenos Aires, Argentina; ^4^ Hospital Municipal de Rehabilitación Respiratoria María Ferrer, Buenos Aires, Argentina; ^5^ CONICET Universidad de Buenos Aires. Instituto de Investigaciones en Microbiología y Parasitología Médica (IMPaM), Buenos Aires, Argentina

**Keywords:** fenofibrate, chronic Chagas disease, inflammation, monocyte subsets, cytokine

## Abstract

Chronic Chagas disease cardiomyopathy (CCC) is the most important clinical manifestation of infection with *Trypanosma cruzi* (*T. cruzi*) due to its frequency and effects on morbidity and mortality. Peripheral blood mononuclear cells (PBMC) infiltrate the tissue and differentiate into inflammatory macrophages. Advances in pathophysiology show that myeloid cell subpopulations contribute to cardiac homeostasis, emerging as possible therapeutic targets. We previously demonstrated that fenofibrate, PPARα agonist, controls inflammation, prevents fibrosis and improves cardiac function in a murine infection model. In this work we investigated the spontaneous release of inflammatory cytokines and chemokines, changes in the frequencies of monocyte subsets, and fenofibrate effects on PBMC of seropositive patients with different clinical stages of Chagas disease. The results show that PBMC from Chagas disease patients display higher levels of IL-12, TGF-β, IL-6, MCP1, and CCR2 than cells from uninfected individuals (HI), irrespectively of the clinical stage, asymptomatic (Asy) or with Chagas heart disease (CHD). Fenofibrate reduces the levels of pro-inflammatory mediators and CCR2 in both Asy and CHD patients. We found that CHD patients display a significantly higher percentage of classical monocytes in comparison with Asy patients and HI. Besides, Asy patients have a significantly higher percentage of non-classical monocytes than CHD patients or HI. However, no difference in the intermediate monocyte subpopulation was found between groups. Moreover, monocytes from Asy or CHD patients exhibit different responses upon stimulation *in vitro* with *T. cruzi* lysates and fenofibrate treatment. Stimulation with *T. cruzi* significantly increases the percentage of classical monocytes in the Asy group whereas the percentage of intermediate monocytes decreases. Besides, there are no changes in their frequencies in CHD or HI. Notably, stimulation with *T. cruzi* did not modify the frequency of the non-classical monocytes subpopulation in any of the groups studied. Moreover, fenofibrate treatment of *T. cruzi*-stimulated cells, increased the frequency of the non-classical subpopulation in Asy patients. Interestingly, fenofibrate restores CCR2 levels but does not modify HLA-DR expression in any groups. In conclusion, our results emphasize a potential role for fenofibrate as a modulator of monocyte subpopulations towards an anti-inflammatory and healing profile in different stages of chronic Chagas disease.

## Introduction

The acute phase of *Trypanosoma cruzi* (*T. cruzi*) infection is characterized by the presence of parasites in the host bloodstream that disseminate to the heart and other organs. This promotes a severe inflammatory response with recruitment of mononuclear cells, activation of resident macrophages, and release of pro-inflammatory mediators. This response is associated with parasite persistence in the heart and other tissues, due to the fact that the immune response is not efficient to wipe out the infection, leading to lifelong infection (Trachtenberg and Hare, 2017). Therefore, it goes forward to a chronic stage with a wide spectrum of manifestations, ranging from minor myocardial involvement to chronic Chagas disease cardiomyopathy (CCC) in which the tropism of the parasite for cardiac tissue constitutes one of the factors that lead to cardiac pathology (Tanowitz et al., 2015). Moreover, inflammatory processes also promote heart muscle fibrosis. Consequently, infected individuals may undergo heart chamber remodeling, congestive heart failure, and eventually death. Likewise, the persistence of activated macrophages in the tissues may create an inflammatory microenvironment that, in turn, contributes to developing tissue damage during the course of these pathological processes (Rőszer et al., 2013).

It has been described that, in response to infection with *T. cruzi*, cardiomyocytes and macrophages release nitric oxide (NO), cytokines and chemokines that are important to control parasite proliferation (Petray et al., 1994). However, the excess of these mediators generates harmful effects, contributing to the pathogenesis of chronic CCC (Machado et al., 2000; Gutierrez et al., 2009; Hovsepian et al., 2011; Penas et al., 2013).

Monocytes are heterogeneous, multifunctional cells that participate in cellular processes, namely, tissue repair and regeneration during heart diseases (Apostolakis et al., 2010). Advances in pathophysiology demonstrate that some subpopulations of myeloid cells contribute to cardiac homeostasis (Bajpai et al., 2018). Monocytes may differentiate into tissue-resident macrophages in specific microenvironmental conditions (Guilliams and Scott, 2017). Currently, the circulating human monocytic cells can be divided into subpopulations based on the surface expression of CD14 (a cell co-receptor for LPS) and CD16 (the low-affinity IgG receptor). They are further divided into three major subsets: a high percentage of monocytes, named classical monocytes, are CD14^++^ CD16^−^ but, to a lesser extent we find two other subpopulations, such as CD14^++^ CD16^+^, intermediate monocytes, and CD14^+^ CD16^++^, which are non-classical monocytes (Wong et al., 2011). Human peripheral blood monocytes are also defined by the expression of the cell surface markers CD64 (Fcγ RI) and the chemokine receptor CD192 (also known as CCR2, a key mediator of monocyte migration) whose most prominent role is the mobilization of monocytes under physiologic and also inflammatory conditions. Besides, monocyte subpopulations can be characterized according to different levels of human leukocyte antigen D related (HLA-DR) (Shi, 2014).

CCR2 was first identified on monocytes, which constitutively express the receptor, and is dowregulated after differentiation into macrophages (Fantuzzi et al., 1999). Particularly, CCR2 plays important roles in tissue recruitment and transmigration of monocytes through the endothelial layer under inflammatory conditions. After myocardial injury, CCR2^-^ macrophages promote the regeneration of cardiac tissue and functional recovery of the heart, through expansion of the coronary vasculature and physiological proliferation of cardiomyocytes (Lavine et al., 2014; Leid et al., 2016). The resident macrophage population was shown to expand in response to cardiac injury by participating in the immune surveillance of this tissue, which raises important questions about the fate and function of macrophages during the development of heart failure (Epelman et al., 2014; Heidt et al., 2014). On the other hand, the recruitment of monocytes, their differentiation into macrophages and their activation have a causal role in ventricular dysfunction (Hulsmans et al., 2018) and fibrosis (Sica et al., 2014; Satoh et al., 2017).

Like in many other situations, monocytes/macrophages as innate immune cells recognize *T. cruzi* pathogen-associated molecular patterns (PAMPs) and activate lymphocytes and the adaptive immune response during Chagas disease (Teixeira et al., 2011; Andrade and Gollob KJ, 2014). It has been shown that individuals infected with *T. cruzi*, with severe heart disease, display a profile of subsets of monocytes that suggests a more pronounced inflammatory environment compared with patients with heart failure unrelated to *T. cruzi* infection (Pérez-Mazliah et al., 2018). Besides, it was reported that the intermediate monocyte subpopulation is associated with CCC (Gómez-Olarte et al., 2019). On the other hand, it has been proposed that monocytes play a role as immunoregulators in asymptomatic Chagas disease patients by activating lymphocytes and, thus, the adaptive immunity through the expression of the co-stimulatory molecules CD80 and CD86. Indeed, the expression of the latter was associated with a higher frequency of Treg cells in asymptomatic individuals (Pinto et al., 2018).

Peroxisome proliferator-activated receptors (PPARs), members of the steroid hormone receptor superfamily, are ligand-dependent nuclear transcription factors. Fenofibrate, a PPAR-α ligand, is a third-generation fibric acid derivative currently used clinically as a hypolipidemic agent to lessen the risk of atherosclerosis (Ling and Luoma, 2013). More than two decades ago, it was shown that PPARs and their ligands can repress inflammatory genes in activated monocytes and macrophages (Ricote et al., 1998; Tontonoz et al., 1998). However, the role of the PPARα receptors and their ligands on cardiac remodeling, repair and functionality exerted by monocytes/macrophages in the context of infection with *T. cruzi* has not been extensively studied. The efficacy of PPARα agonists, including fenofibrate, as regulators of inflammation and remodeling of the extracellular matrix of the heart has been reported (Lockyer et al., 2010). Fenofibrate has been shown to be able to prevent cardiac inflammation and fibrosis in diabetic mice (Zhang et al., 2016). Furthermore, it has been shown to exert cardioprotective effects against various cardiac disorders, namely, *in vivo* models of cardiac hypertrophy produced by pressure overload (Zou et al., 2013) or experimental autoimmune myocarditis in rats (Cheng et al., 2016) and also in patients with systolic dysfunction (Yin et al., 2013) or experimental myocardial infarction (Garg et al., 2016). Due to its ability to prevent interstitial and perivascular fibrosis in kidney, liver, lung and heart in different experimental models, fenofibrate has recently been proposed as a potential antifibrotic agent (McVicker and Bennett, 2017).

In previous studies, our group developed an experimental model of Chagas disease, in which mice were sequentially infected with two *T. cruzi* strains, which differ in genetic background and lethality, leading to clear signs of left ventricular dysfunction. In this model, we show that fenofibrate controls inflammation, prevents fibrosis, and improves heart function (Cevey et al., 2017). Furthermore, PPAR agonists contribute to neovascularization and redirect pro-inflammatory to healing macrophages in experimental trypanosomiasis (Penas et al., 2013; Penas et al., 2015; Cevey et al., 2016; Garg et al., 2016; Penas et al., 2016; Cevey et al., 2017; McVicker and Bennett, 2017; Penas et al., 2017; Rada et al., 2020).

Consequently, the aim of this work was to characterize the monocyte populations of patients in different phases of CCC and to study the effect of fenofibrate on these cells in culture. This would allow identifying possible therapeutic targets promoting fenofibrate as a coadjuvant to anti-parasitic treatment.

## Materials and Methods

### Ethics Statement

Informed consent was signed by each subject. The study protocol is in line with the ethical guidelines of the Declaration of Helsinki and was approved by the Ethics Committee of the “Hospital General de Agudos Dr. Cosme Argerich” and of the “Hospital Municipal de Rehabilitación Respiratoria María Ferrer”, Buenos Aires, Argentina.

### Study Cohort

Subjects were recruited at the Cardiology Department of both Hospital General de Agudos Dr. Cosme Argerich and Hospital Municipal de Rehabilitación Respiratoria MaríaFerrer (Ciudad Autónoma de Buenos Aires, Argentina).

#### Inclusion Criteria

Men and women between 18 and 60 years, with positive serology for Chagas disease were included. Each seropositive participant underwent a clinical and cardiological evaluation to determine the clinical stage of the disease. The classification of patients was carried out according to the Chagas Consensus (Healthy or Chagas Stage 0, I, II, and III) in accordance with the criteria of the Argentine Society of Cardiology (Mitelman, 2011). In this work and consistent with this classification, we named the different groups according to the absence of symptoms (Asy) or the presence of any cardiac damage (CHD). Control group included healthy individuals (HI), men and women between 18 and 60 years, with negative serology for Chagas disease. None of the subjects should have co-morbidities at the time of sample collection, nor have received previous treatment for Chagas disease nor with lipid-lowering agents from the group of fibrates or statins.

### Peripheral Blood Mononuclear Cells (PBMC) Isolation

Whole blood (10 to 15 ml) was collected from participants by venipuncture into heparinized tubes (Vacutainer, BD Biosciences). Plasma was obtained by centrifugation and stored at −80°C. Peripheral blood mononuclear cells (PBMC) were isolated by Ficoll–Paque™ PLUS density gradient centrifugation (GE Healthcare, Amersham, Sweden). PBMC were washed twice and suspended in complete culture medium: RPMI 1640 (Invitrogen Life Technologies, Grand Island, NY, USA) supplemented with fetal bovine serum (FBS) 10% (Internegocios S.A., Argentina) and antibiotics (50 μg/ml of PenStrep^®^). All experiments were performed using freshly isolated PBMC.

### *T. cruzi* Culture and Lysate

Vero cells were cultured in cell culture flasks of 175 cm^2^ with RPMI supplemented with 10% fetal bovine serum (FBS), 100 IU/ml Penicillin, 0.1 mg/ml Streptomycin and 2 mM L-glutamine. When culture reached an approximate 50% confluence, it was infected with parasites of the RA strain of *T. cruzi.* After 6 h, the cells were washed with fresh culture medium to remove non-infective parasites and incubated at 37°C for 48 h.

On day 5 post-infection (dpi), trypomastigotes were harvested from the supernatant. The culture medium was collected, two washes were performed with cold PBS and then it was centrifuged at 18,000×*g* at 4°C for 5 min. The parasite pellet was stored at −80°C. After one month of collection, all sediments were pooled and lysed to obtain trypomastigote proteins. Briefly, sediments were resuspended in lysis buffer (PBS, 10 µM E-64 and 3 µg/ml protease inhibitor) and subjected to 3 freeze/thaw cycles (−80°C/room temperature) of 30 min each. Then, it was incubated overnight at −80°C and centrifuged at 17,000×*g* at 4°C for 10 min. Supernatant was collected, and the protein concentration was quantified by the Bradford method using a commercial protein assay (Bio-Rad, USA) and bovine serum albumin (BSA) (Sigma-Aldrich Co, USA) as a standard (Kruger, 1994) as described previously by our group (Cevey et al., 2019; Penas et al., 2020; Rada et al., 2020).

### *In Vitro* Treatments

According to the experiment, cells were pre-treated for 15 min with 100 µM Fenofibrate^®^ (Daunlip^®^, Montpellier S.A, Argentina. PubChem Compound Database CID = 3339, Fen) resuspended in PBS (Cevey et al., 2017). Then, cells were stimulated or not with *T. cruzi* lysate (10 µg/ml) for 20 h.

### Flow Cytometry

This experiment included 17 healthy individuals, 7 asymptomatic patients with positive serology for Chagas disease and 9 with Chagas heart disease. Cells from all experimental groups were cultured for 16–20 h after treatment.

PBMC were stained with LIVE/DEAD™ fixable dye (Invitrogen) at room temperature for 15 min and labeled with the following antibodies at 4°C for 30 min: CD14 (#E-AB-F1209C, Elabscience), CD16 (#E-AB-F1005M, Elabscience), HLA-DR (#E-AB-F1111H, Elabscience), and CCR2 (#357209, Elabscience). Then, cells were washed, fixed and acquired using a FACS Canto (Becton Dickinson). Post-acquisition analysis was performed using FlowJo version 10 software (FlowJo LLC, Ashland, Oregon, USA). In all cases, isotype-matched mAb were used as controls.

#### Gating Strategies

Peripheral blood mononuclear cells (PBMC) were gated based on forward scatter (FSC) and side scatter (SSC) parameters. After excluding doublets and debris using FSC-Width *vs*. FSC-Area, the strategy used to differentiate the three subsets of monocytes was based on CD14 and CD16 expression: classical (CD14high/CD16neg), intermediate (CD14high/CD16pos) and non-classical (CD14low/CD16pos) monocytes. Then, in both total monocytes and subpopulations, the percentage (%) and mean fluorescence intensity (MFI) of the membrane markers CCR2 and HLA-DR was calculated. The MFI was calculated as the geometric mean of the expression.

### RNA Purification

Total RNA was obtained from PBMC using Quick-zol reagent (Kalium Technologies, Argentina), treated with RQ1 RNase-Free DNase (PromegaCo., USA). Total RNA was reverse-transcribed using M-MLV Reverse Transcriptase (Promega Co., USA), according to manufacturer’s instructions as described previously by our group (Cevey et al., 2019; Penas et al., 2020; Rada et al., 2020).

### Quantitative Reverse Transcription Polymerase Chain Reaction (RT-qPCR)

**Figure 1:**
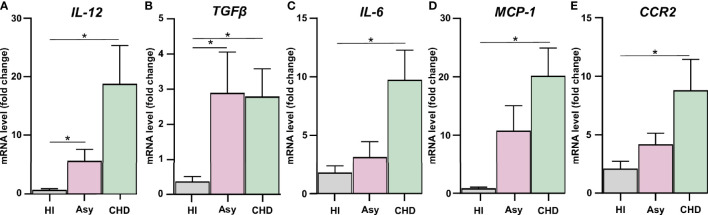
Assessment of the expression of pro-inflammatory mediators and CCR2. Expression of IL-12 **(A)**, TGF-β **(B)**, IL-6 **(C)**, MCP-1 **(D)**, and CCR2 **(E)** were determined by RT-qPCR in PBMC of healthy individuals (HI), seropositive asymptomatic (Asy) or Chagas heart disease (CHD) after 48 h of culture. mRNA expression was analyzed and normalized against β-Actin. Results are expressed as the mean of 3 independent experiments. Differences between groups were analyzed by Kruskal–Wallis test (mean ± SEM) followed by Dunn’s *post hoc* test. *P < 0.05. PBMC of Asy or CHD vs. PBMC of HI.

**Figure 2:**
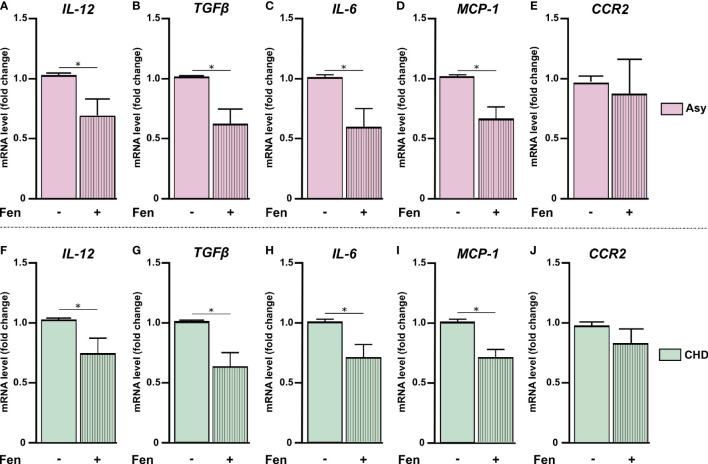
Fenofibrate modulates pro-inflammatory mediators’ expression. PBMC were treated *in vitro* or not with 100 µM of fenofibrate. After 48 h, IL-12, TGF-β, IL-6, MCP-1, and CCR2 mRNA was measured in asymptomatic (Asy) **(A-E)** and Chagas heart disease patients (CHD) **(F-J)**. mRNA levels were determined by RT-qPCR and normalized against β-Actin. Results are expressed as mean of 3 independent experiments. Differences between fenofibrate-treated PBMC were analyzed using the Wilcoxon test for paired samples and are shown as the mean of the experiments ± SEM. *P < 0.05. Fen-treated PBMC vs. untreated PBMC.

mRNA expression was performed using 5× HOT FIREPOL EVAGREEN qPCR (SolisBioDyne, Estonia) in a StepOnePlus Real-Time PCR System. Parameters were: 52°C for 2 min, 95°C for 15 min, and 40 cycles at 95°C for 15 s, specific Tm °C for 30 s and 72°C for 1 min. Normalization was carried out using β-Actin mRNA. Quantification was performed using the comparative threshold cycle (Ct) method, as all the primer pairs (target gene/reference gene) were amplified using comparable efficiencies (relative quantity, 2^−ΔΔCt^) (Schmittgen and Livak, 2008; Bustin et al., 2009). To evaluate the expression of inflammatory mediators in the PBMC of both the asymptomatic (Asy) and cardiac (CHD) patient groups, the PBMC of healthy individuals (HI) were used as reference control (**Figure 1**). On the other hand, to study the effects of fenofibrate treatment *in vitro*, on the expression of inflammatory mediators in PBMC of Asy and CHD patients, PBMC samples from each patient not treated with fenofibrate were taken as reference controls (**Figure 2**). mRNA expression of IL-12, TGF-β, IL-6 and MCP-1 was measured in 20 healthy individuals, 13 asymptomatic patients and 28 cardiac patients. CCR2 mRNA expression was measured in 6 healthy individuals, 4 asymptomatic patients and 5 cardiac patients.

### Primer Sequences

**Table:** 

	Forward (5’-3’)	Reverse (5’-3’)
IL-12	CTCCTGGACCACCTCAGTTT	TGGTGAAGGCATGGGAACAT
TGF-β	ATGGAGAGAGGACTGCGGAT	TGGTCCCCTGTCCTATGA
IL-6	TATTAGAGTCTCAACCCCCAATAAA	ACCAGGCAAGTCTCCTCATT
MCP-1	CTCTCGCCTCCAGCATGAAA	CTTGAAGATCACAGCTTCTTTGG
CCR2	CATTAGTTGCCCTGTATCTC	ATGCGTCCTTGTTCAATCC
β-Actin	GTGGGGCGCCCCAGGCACCA	CGGTTGGCCTTGGGGTTCAGGGGG

### Statistical Analysis

Different statistical tests were used for this work. To compare baseline cytokine mRNA expression between healthy donors, asymptomatic or Chagas heart disease patients, nonparametric Kruskal–Wallis test, and then the Dunn´s multiple comparisons test were performed. For the study of the effect of fenofibrate in mRNA expression, a non-parametric Wilcoxon test was used for paired samples. Mixed-effects model analysis was performed to analyze differences between experimental groups in flow cytometry assays. The Tukey *post-hoc* test was performed to compare every mean with every other mean. Differences were considered statistically significant when *P <*0.05. All analyses were performed using the Prism 7.0 Software.

## Results

### Cohort Characteristics of Chagas Disease Patients

**Table 1** shows clinical and electrocardiographic findings in the cohort of Chagas disease patients under study. They were classified according to the absence of symptoms (Asy) or the presence of any cardiac damage (CHD) in stage I, II, or III according to severity. Control group included healthy individuals (HI), men and women between 18 and 60 years, with negative serology for Chagas disease. Most patients were born in disease endemic areas with vectorial transmission.

**Table 1:** Clinical details of the study population.

Group^a^	No. of individuals	Age range (median), yr	Born in endemic areas	Clinical Finding(s)^b^	ECG finding(s)^c^
HI	23	40–81 (53)	NA	Normal	Normal/NA
Asy	19	33–75 (53)	18/19	Asymptomatic	Normal
CHD-Stage I	14	44–78 (66,5)	14/14	AV block, AF, PMK, isolated VE, DCM	LAFB, RBBB, PMK, AF
CHD-Stage II	8	31–78 (57)	8/8	NSVT with VEs, VEs, SVT, PPM with ICD, DCM	Repolarization/NA
CHD-Stage III	19	45–69 (58)	19/19	VT with ICD, AVS, AF, PMK, AV block, PPM with ICD, VEs, CHD	RBBB, PPM, LAFB, AF, AV block, LBBB, PMK

a The patients were categorized as follows: healthy individuals (HI), patients with positive serology for Chagas disease but asymptomatic (Asy), and patients with chronic heart disease (CHD). The latter, separated into Stage I if there is an abnormal ECG but a normal chest X-ray, Stage II if the X-ray is also abnormal and Stage III, if there are more symptoms of heart failure.

b The main clinical findings were different arrhythmias: Atrioventricular block (AV block), atrial fibrillation (AF), pacemaker (PMK), ventricular extrasystoles (VE or VEs if frequent), dilated cardiomyopathy (DCM), no sustained ventricular tachycardia (NSVT), supraventricular tachycardia (SVT), permanent pacemaker (PPM), implantable cardioverter defibrillator (ICD), ventricular tachycardia (VT), acute vestibular syndrome (AVS) and coronary heart disease (CHD).

c ECG (electrocardiographic) findings: Left anterior fascicular block (LAFB), right bundle branch block (RBBB) and left bundle branch block (LBBB); data not available (NA).

Endemic areas of patients: Argentinean provinces: Santiago del Estero, Chaco, Salta, Córdoba, Santa Fé, Jujuy, San Juan, Mendoza. Other countries: Bolivia and Paraguay.

### Pro-Inflammatory Mediators’ Expression in PBMC From Chagas Disease Patients

Groups of seropositive patients with different clinical forms for Chagas disease were included in this study, namely, with cardiomyopathy and without evidence of cardiac symptoms, and also healthy individuals. In order to evaluate whether spontaneous release of inflammatory cytokines and chemokines were differentially expressed, cultured PBMC from patients with Chagas disease were analyzed. mRNA levels of IL-12, IL-6, TGF-β, MCP-1, and CCR2 were determined by RT-qPCR, to assess the basal level production of pro-inflammatory mediators. We observed that PBMC from Chagas disease patients displayed a higher level of cytokines than cells from HI, irrespective of the clinical stage of the disease. The expression of IL-12 in PBMC of Asy patients with positive serology for Chagas but without heart disease was higher than HI. However, in patients with CHD its expression was even higher, as shown in **Figure 1A**. A similar result can be observed in the evaluation of TGF-β, since PBMC of both groups of patients show increased levels of this cytokine with respect to the values of healthy individuals (**Figure 1B**). However, when the expression of IL-6, MCP-1, and CCR2 was evaluated, we found that only PBMC from patients with heart disease displayed significantly increased levels of these cytokines in comparison with healthy individuals (**Figures 1C–E**).

### Fenofibrate Reduces Inflammatory Mediator Levels in PBMC From Chagas Heart Disease Patients

We have previously reported that fenofibrate significantly reduces the extension of heart infiltrates and the expression of pro-inflammatory cytokines in a murine model of mixed-stains infection with bloodstream trypomastigotes (Cevey et al., 2017; Rada et al., 2020). In this work, we observed that pro-inflammatory mediators displayed higher levels of expression in cultured PBMC from Chagas disease patients in comparison with those of HI. In order to evaluate whether treatment with fenofibrate was able to promote a reduction of pro-inflammatory cytokines in isolated PBMC *in vitro*, we assessed the levels of mRNA expression of those mediators. We previously demonstrated, in a work by our group and in line with other studies, that 100 µM of fenofibrate is the optimal concentration at which inflammatory mediators are inhibited without affecting the viability of primary cardiomyocyte cultures (Penas et al., 2015; Nahrendorf, 2018). As shown in **Figure 2**, *in vitro* treatment of PBMC from Asy and CHD patients with 100 µM fenofibrate, reduces the levels of IL-12, TGF-β, IL-6, MCP1 in comparison with untreated cells (**Figures 2A–D, F–I**). However, CCR2 expression levels were not significantly modified in Asy or CHD patients (**Figures 2E, J**). Likewise, fenofibrate does not modify the expression of any of the studied cytokines in PBMC from healthy individuals (**Supplementary Figure S1A**).

### Patients With Different Stages of Chagas Disease Display Changes in the Frequencies of Monocyte Subsets

**Figure 3:**
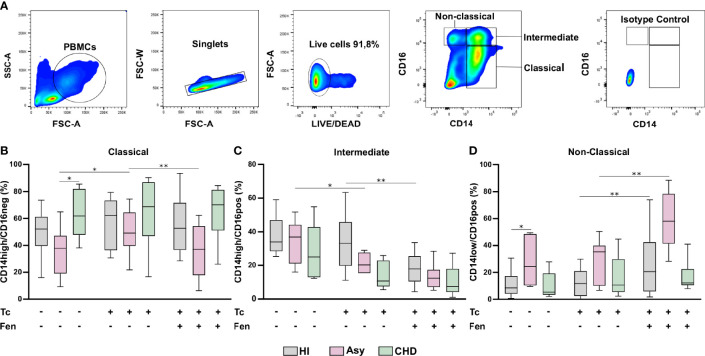
Percentage of monocyte subpopulations in patients with Chagas heart disease (CHD), asymptomatic (Asy) and healthy individuals (HI) with/without *in vitro* treatment. A representative analysis of the gating strategy used in this study to differentiate the three monocyte subpopulations is shown **(A)**. PBMC were stimulated or not with *T. cruzi* lysate (Tc) and treated or not with fenofibrate (Tc + Fen). After 20 h according to CD14 and CD16 expression, they were classified as Classical (CD14high/CD16neg), Intermediate (CD14high/CD16pos) and Non-Classical (CD14low/CD16pos). Percentage of classical **(B)**, intermediate **(C)** and non-classical **(D)** monocytes for HI, Asy and CHD unstimulated, stimulated with Tc lysate and with Tc lysate + Fen treatment. These data were analyzed by fitting a mixed model with a Tukey *post-hoc* test and the results are expressed as the mean of the experiments ± SEM. *P < 0.05. CHD vs. Asy; Asy vs. HI. *P < 0.05. Tc vs. untreated; **P < 0.01. Tc + Fen vs. Tc.

Different subpopulations of monocytes are involved in the progression of Chagas disease cardiomyopathy. To determine whether certain monocyte subsets were particularly expressed in Chagas disease patients, according to the disease stage, the monocyte population in whole PBMC from HI, Asy and CHD patients was characterized by flow cytometry analysis (FACS), according to the expression of CD14 and CD16, as classical (CD14^high^ CD16^neg^), intermediate (CD14^high^ CD16^pos^) and non-classical (CD14^low^ CD16^pos^) (**Figure 3A**). **Figures 3B–D** show the effects of *T. cruzi* stimulation and treatment of PBMC with Fen, on the percentage of classical, intermediate, and non-classical monocytes from uninfected, asymptomatic and Chagas heart disease patients. The results depicted in **Figure 3B** in *T. cruzi* unstimulated and untreated cells, show a significantly higher level of classical monocytes in CHD patients in comparison with Asy. However, under the same conditions the subpopulation of intermediate monocytes, CD14^high^ CD16^pos^, does not show differences between CHD and Asy or HI (**Figure 3C**). Notably, *T. cruzi* unstimulated and untreated cells from Asy patients have a significantly higher percentage of non-classical monocytes than HI (**Figure 3D**).

### *In Vitro* Stimulation With *T. cruzi* and Fenofibrate Treatment Changes the Prevalence of Monocyte Subsets According to the Stage of the Disease

We sought to determine whether monocytes from Asy or CHD patients exhibit different responses against the parasite. To this aim, purified PBMC were stimulated *in vitro* with *T. cruzi* lysates (10 μg/ml) for 24 h. **Figure 3B** shows that stimulation with lysates significantly increases the percentage of CD14^high^ CD16^neg^, classical monocytes, of the Asy group, but does not change their frequencies in CHD or HI groups. On the other hand, stimulation with *T. cruzi* lysates decreases the percentage of CD14^high^ CD16^pos^, Intermediate monocytes, in Asy patients, and a trend to decreased frequencies is also observed in CHD patients (**Figure 3C**). However, no changes were observed in the percentage of this monocyte subset in HI (**Figure 3C**). Notably, the stimulation with *T. cruzi* did not modify the frequency of non-classical subpopulation, CD14^low^CD16^pos^, monocytes in any of the groups studied (**Figure 3D**).

Fenofibrate pre-treatment of *T. cruzi-*stimulated monocytes exerted a modulatory effect, decreasing the percentage of classic monocytes in the Asy population (**Figure 3B**). **Figure 3C** shows that fenofibrate decreases the frequency of CD14^high^CD16^pos^ cells in HI, but we only observed a trend to decrease this cell subset in Asy and CHD patients. Interestingly, fenofibrate significantly increased the percentage of non-classical cells in the HI and Asy groups but this was not modified in the CHD group (**Figure 3D**). Of note, fenofibrate alone does not modify any monocyte subpopulation in HI, Asy, and CHD (**Figure S1B**).

### *In Vitro T. cruzi* Stimulation and Fenofibrate Treatment Modify CCR2 and HLA-DR Expression in Monocyte Subsets

**Figure 4:**
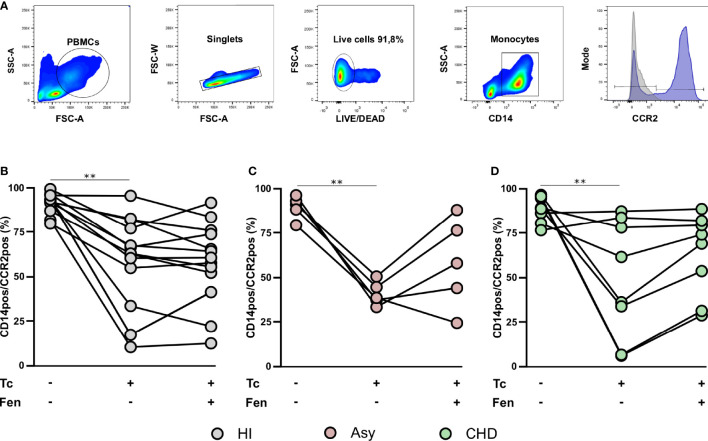
*T. cruzi* stimulation and fenofibrate treatment modify CCR2 in CD14pos cells. Percentage of CCR2 was determined in basal CD14pos cells after 20 h of *T. cruzi* lysate (Tc) stimulation or fenofibrate (Tc + Fen) treatment. Monocytes were selected based on FSC and SSC. After excluding doublets and debris, live cells were selected, monocytes were classified by CD14 positive staining. Representative histograms show the number of events and expression level of CCR2 **(A)**. The percentages of CD14pos/CCR2pos monocytes are shown in healthy individuals (HI) **(B)**, asymptomatic (Asy) **(C)** and patients with Chagas heart disease (CHD) **(D)**, where each patient is represented by a circle. The results are shown as the mean of the experiments ± SEM. These data were analyzed by fitting a mixed model with a Tukey *post-hoc* test. **P < 0.01. Tc vs. untreated.

The most prominent role of CCR2 is believed to be in the mobilization of monocytes under physiologic and also inflammatory conditions. With the purpose to establish the expression of CCR2 on total monocytes (CD14^+^ cells), according to clinical status, PBMC from CHD, Asy, and HI groups were stained with CD14 and CCR2 and analyzed by FACS (**Figure 4A**). The results show high basal levels of CCR2 in CD14^+^CCR2^+^ monocytes of all groups (**Figures 4B-D**). When CD14^+^CCR2^+^ cells were stimulated with *T. cruzi* lysates, its percentage in Asy and CHD patients decreased significantly, and also in healthy controls (**Figures 4B–D**). Furthermore, especially in Asy, the pretreatment with fenofibrate of monocytes stimulated with *T. cruzi* induces a trend to restore the basal levels of CD14^+^CCR2^+^ cells (**Figure 4C**). Besides, that fenofibrate does not modify CCR2^+^ levels in unstimulated CD14^+^ cells (**Supplementary Figure S1C**).

**Figure 5:**
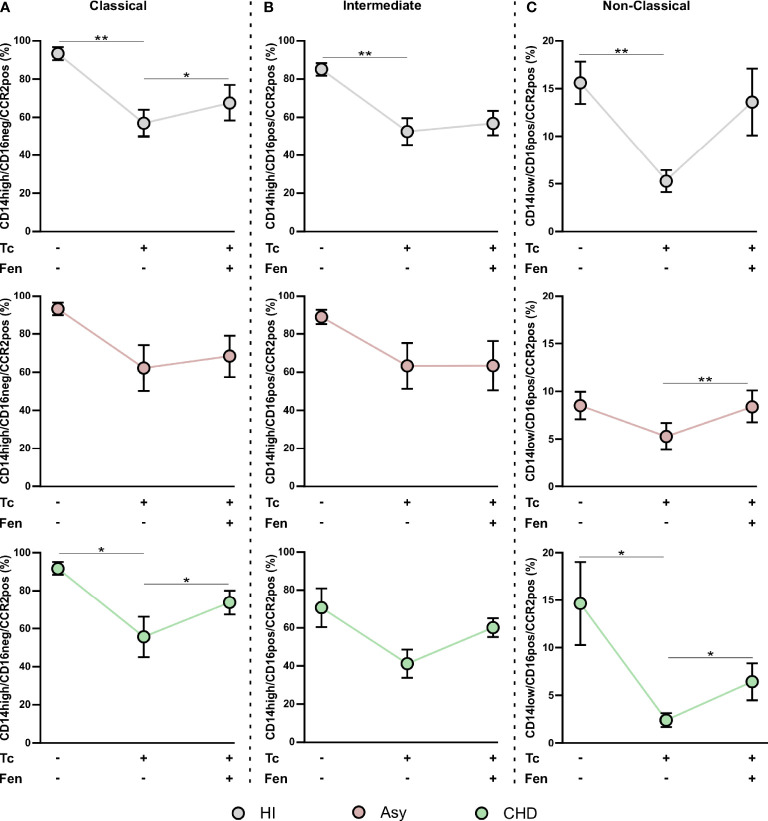
*T. cruzi* stimulation and fenofibrate treatment modify CCR2 in monocyte subpopulations. Percentage of CCR2^+^ cells was determined in PBMC stimulated or not with *T. cruzi* lysate (Tc) and treated or not with fenofibrate (Tc + Fen) after 20 h, according to CD14 and CD16 expression. It shows the percentage of classical (CD14high/CD16neg) **(A)**, intermediate (CD14high/CD16pos) **(B)** and non-classical (CD14low/CD16pos) **(C)** monocytes with CCR2^+^ expression. The results are shown as the mean of the experiments ± SEM. These data were analyzed by fitting a mixed effect model with a Tukey *post-hoc* test. *P < 0.05; **P < 0.01.

**Figure 6:**
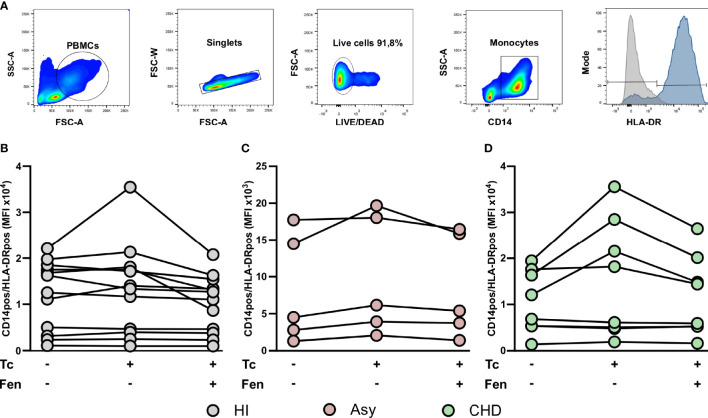
HLA-DR expression in monocyte. The mean fluorescence intensity (MFI) of HLA-DR was determined in basal CD14pos cells after 20 h of *T. cruzi* lysate (Tc) stimulation or fenofibrate (Tc + Fen) treatment. Monocytes were selected based on FSC and SSC. After excluding doublets and debris, live cells were selected, monocytes were classified by CD14 positive staining. The mean fluorescence intensity (MFI) of HLA-DR was calculated both in total monocytes **(A)**. It shows the mean fluorescence intensity (MFI) of CD14pos/HLA-DRpos monocytes in healthy (HI) **(B)**, asymptomatic (Asy) **(C)** and chronic Chagas disease (CHD) patients **(D)**, where each patient is represented by a circle. The results are shown as the mean of the experiments ± SEM. These data were analyzed by fitting a mixed effect model with a Tukey *post-hoc* test.

When CCR2 expression was analyzed in the different monocyte subpopulations, we observed that the percentage of CCR2^+^ cells in HI and CHD decreased in the classical monocyte subpopulation stimulated with *T. cruzi* lysates. Furthermore, fenofibrate treatment restored the baseline level only in monocytes from CHD patients and HI (**Figure 5A**). The intermediate monocyte subpopulation showed a decrease in its expression upon stimulation with *T. cruzi*, which was only significant in monocytes from HI. However, fenofibrate did not restore the decreased expression of CCR2 (**Figure 5B**). Regarding the subpopulation of non-classical monocytes, the stimulation with *T. cruzi* significantly decreased the percentage of cells expressing CCR2 in the CHD and HI groups, although only with a trend in Asy patients. Treatment with fenofibrate significantly increased the percentage of CCR2 in Asy and CHD as shown in **Figure 5C**. In order to determine the expression of HLA-DR in different disease stages, PBMC of the three experimental groups were stained with CD14 and HLA-DR (**Figure 6A**). **Figures 6B, C** show no significant differences between cells stimulated *in vitro* with *T. cruzi* lysates in comparison with unstimulated cells, in both Asy and HI. However, there is a trend to increased expression of HLA-DR with *T. cruzi* stimulation in CHD patients, according to its mean fluorescence intensity (MFI) (**Figure 6D**). As in the case of CCR2, fenofibrate alone did not modify the increased expression of HLA-DR in unstimulated and treated cells (**Supplementary Figure S1C**). This was also observed in cells from Asy and HI stimulated with *T. cruzi*. However, fenofibrate shows a clear tendency to inhibit the increased HLA-DR expression of PBMC from CHD upon stimulation with *T. cruzi* (**Figure 6D**).

**Figure 7:**
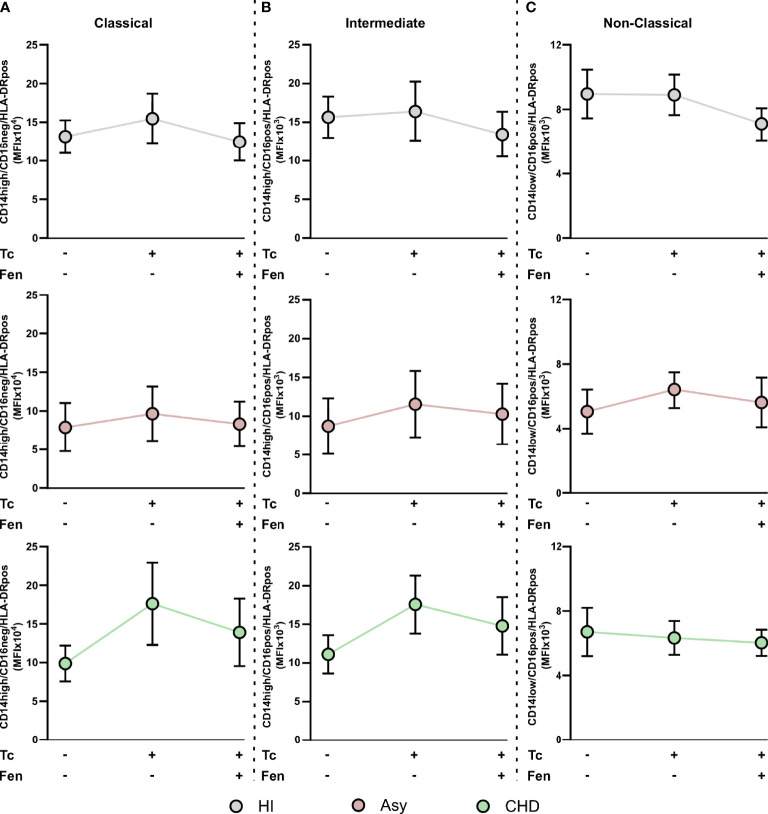
HLA-DR expression in *T. cruzi* stimulated and fenofibrate treated monocyte subpopulations. The mean fluorescence intensity percentage of HLA-DR^+^ cells was determined in PBMC stimulated or not with *T. cruzi* lysate (Tc) and treated or not with fenofibrate (Tc + Fen) after 20 h, according to CD14 and CD16 expression. It shows the mean fluorescence intensity (MFI) of classical (CD14high/CD16neg) **(A)**, intermediate (CD14high/CD16pos) **(B)** and non-classical (CD14low/CD16pos) **(C)** monocytes with HLA-DR^+^ expression. The results are shown as the mean of the experiments ± SEM. These data were analyzed by fitting a mixed effect model with a Tukey *post-hoc* test.

When we studied HLA-DR expression in the classical monocyte subpopulation, we observed that stimulation with *T. cruzi* tends to raise the MFI of monocytes from CHD patients and treatment with fenofibrate displays a trend to restore it to basal levels (**Figure 7A**). We found that both stimulation with *T. cruzi* lysates and fenofibrate treatment did not modify HLA-DR expression in intermediate or non-classical monocyte subpopulations, as shown in **Figures 7B, C**. Besides, fenofibrate neither modifies CCR2^+^ nor HLA-DR levels in unestimulated monocytes subsets of CHD, Asy or HI (**Supplementary Figure S2A**).

## Discussion

Monocytes are heterogeneous and multifunctional cells. As components of the innate immune response, they participate not only in inflammation and fibrosis, but also in tissue repair and regeneration during heart diseases (Apostolakis et al., 2010). Consequently, inflammatory cells, such as monocytes, are increasingly being considered as potential drug targets for the treatment of different heart conditions (Nahrendorf, 2018). Regarding Chagas disease, while the relevance of parasite persistence as a trigger of tissue damage is currently acknowledged in the development of CCC, the role of the different components of the inflammatory response remains unclear (Zhang and Tarleton, 1999; Lopez et al., 2018; Wesley et al., 2019).

In the present work, we first determined the spontaneous expression of pro-inflammatory mediators, such as MCP-1 and its receptor CCR2, and IL-12, TGF-β, IL-6 in cultured PBMC from seropositive patients with different clinical forms of Chagas disease. The results show that PBMC from CHD patients displayed a significantly higher expression level of these mediators than cells from uninfected individuals. Moreover, in Asy patients we observed a significantly increased expression of IL-12 and TGF-β, and a trend to increase in the other cytokines evaluated (**Figure 1**). These results are in consonance with those found by other authors in neutrophils and monocytes for similar groups of patients (Souza et al., 2004; Campi-Azevedo et al., 2015; Medeiros et al., 2017; Pinto et al., 2018).

Recently, Dey et al. showed that mouse CD11c^+^ classical dendritic cells, but not CD11b^+^ Ly6c^+^ inflammatory monocytes, are the source of IL-6 required for the expansion of protective Th17 cells against drug-resistant *Leishmania donovani* (Dey et al., 2020). Previous studies by Stäger et al. also reported that IL-6 deficiency was associated with the expansion of IL-10-producing Treg cells, while expansion was not observed in IL-12p40-deficient mice, stressing the role of IL-6 in the control of infection (Stäger et al., 2006). Regarding our results, it must be noted however, that the actual source of these, and also the other cytokines was not investigated. Independently of this fact, it is clear that both IL-12 and IL-6 are associated with a more severe outcome, since they were particularly elevated in symptomatic patients in comparison with asymptomatic ones or healthy controls. Since in the context of parasite persistence in the tissues, *T. cruzi* induces a substantial increase in pro-inflammatory mediators and reactive oxygen and nitrogen species (Cevey et al., 2017; Penas et al., 2017), this scenario would favor tissue damage and contributes to the clinical outcome observed in symptomatic patients with Chagas disease. For this reason, it is desirable to find an anti-inflammatory therapy that might be useful as a coadjuvant of the antiparasitic treatment to preclude the onset of heart damage during the course of infection.

Previous studies from our group showed that PPAR agonists, such as fenofibrate, a potent hypolipidemic drug, also bears anti-inflammatory properties in the context of experimental Chagas disease (Cevey et al., 2017; Rada et al., 2020). In this work we demonstrated that *in vitro* treatment with fenofibrate significantly reduces the expression of pro-inflammatory mediators, in PBMC (**Figure 2**). These findings are in line with those of Krysiak et al., in which fenofibrate decreased the release of TNFα, IL-1β, IL-6, and MCP-1 by human monocytes. Those effects were accompanied by a decrease in plasma C-reactive protein levels, which could be clinically relevant in the prevention of vascular complications (Krysiak et al., 2011). In this regard, we demonstrated that fenofibrate controls inflammation, prevents fibrosis, contributes to neovascularization and improves left ventricular function, in an experimental murine model of Chagas disease (Cevey et al., 2017). Moreover, in another work, we showed that this occurs through IL-10-dependent and -independent mechanisms (Rada et al., 2020). These anti-inflammatory and protective effects of fenofibrate have been also evaluated in models of autoimmune myocarditis (Cheng et al., 2016), skeletal muscle inflammation (Dai et al., 2016), and cardiac ischemia/reperfusion models (Sugga et al., 2012).

In this work, we studied the monocyte population of patients with different stages of Chagas disease. Our results show that patients with CHD have a significantly higher percentage of classical monocytes. Besides, we determined that there are no significant differences between HI, Asy, or with CHD individuals in the intermediate monocyte subpopulation. Interestingly, we found that Asy patients have a significantly higher percentage of non-classical monocyte subpopulation (**Figure 3**), suggesting they are in an alert and patrolling state. In this sense, the work of Cros et al. demonstrated that human non-classical monocytes exhibited endothelial crawling behavior after adoptive transfer to mice (Cros et al., 2010). Our results are in line with a work by Pérez-Mazliah et al. who showed increased levels of non-classical monocytes in *T. cruzi*-infected individuals with mild or no signs of cardiac disease, as well as in patients suffering from dilated cardiomyopathy unrelated to *T. cruzi* infection. In contrast, they also showed that the monocyte profile in *T. cruzi*-infected individuals with severe cardiomyopathy was slanted to the classical and intermediate subsets (Pérez-Mazliah et al., 2018). Consequently, *in vitro* experiments showed that CD16^+^ monocytes have higher mobility than their CD16^-^ counterparts (Randolph et al., 2002). This behavior suggests that non-classical monocytes are constantly inspecting the endothelium for signs of inflammation or damage and preparing to rapidly transmigrate (Wong et al., 2012). Another study attributed the differences in the characterization of the monocyte subpopulations, partly due to the fact that individuals infected with parasites of different discrete typing units (DTU) triggers different immunological impact which could influence the progression of the disease (Passos et al., 2015).

In our work, we evaluated whether *in vitro* treatment with fenofibrate promotes changes in monocyte subpopulations. We determined that *T. cruzi* increased the percentage of classical monocytes of Asy patients, while fenofibrate treatment inhibited this effect. In contrast, there were no changes in the classical monocyte population from patients with CHD. On the other hand, *T. cruzi* produced a significant decrease in the intermediate population of Asy patients that was not modified by fenofibrate. Notably, fenofibrate significantly increased the percentage of non-classical monocytes in Asy patients, suggesting that this treatment promotes a repairing and patrolling behavior. Also, it should be noted that both *T. cruzi* and fenofibrate were not able to significantly modify the percentage of any of the monocyte subpopulations of patients with CHD (**Figure 3**).

CCR2 is a chemokine receptor involved in monocyte mobilization and plays a key role in extravasation and transmigration of monocytes under inflammatory conditions (Conductier et al., 2010; Chu et al., 2014). MCP-1, ligand of CCR2, is a potent monocyte activator that is abundantly expressed in various pathological conditions. It has been demonstrated that the loss of MCP-1 by targeted gene disruption is sufficient to impair monocyte trafficking in several inflammation models (Lu et al., 1998). Studies carried out with CCR2^−/−^ mice clearly demonstrated that this receptor exhibits functions as a major mediator of macrophage recruitment and trafficking in host defense to bacterial and parasitic infections (Hardison et al., 2006). Our results clearly show that when monocytes are stimulated with *T. cruzi* lysates, the percentage of CCR2 decreases in all the groups (**Figure 4**). It has been postulated that this might occur due to the fact that during chemotaxis, CCR2^+^ expressed on monocytes internalizes with the bound chemoattractant, but cycles rapidly back to the plasma membrane to maintain high responsiveness (Volpe et al., 2012; Zhao et al., 2019). Notably, fenofibrate tends to restore baseline levels of CCR2 in cells stimulated with *T. cruzi* (**Figure 4**). It has been described that CCR2^−^ and CCR2^+^ macrophages have distinct functions in the heart. CCR2^−^ macrophages are involved in various forms of tissue remodeling such as coronary development, postnatal coronary growth, and cardiac regeneration (Lavine et al., 2014; Leid et al., 2016). Moreover, some studies revealed that after neonatal cardiomyocyte injury, CCR2^−^ macrophages contribute to cardiac tissue regeneration and functional recovery of the heart, expanding the coronary vasculature, cardiomyocyte proliferation, and physiological cardiomyocyte hypertrophy. Particularly, in the pediatric mouse heart, the absence of CCR2^−^ macrophages demonstrated poor regenerative capacity (Epelman et al., 2014; Lavine et al., 2014; Leid et al., 2016). However, analysis of heart transplant recipients in humans revealed that CCR2^−^ macrophages are a tissue-resident population exclusively replenished through local proliferation, whereas CCR2^+^ macrophages do it through monocyte recruitment and proliferation. In patients with heart failure, CCR2^+^ macrophage abundance is associated with left ventricular remodeling and systolic function (Bajpai et al., 2018).

Although fenofibrate restored CCR2^+^ in classical subpopulation of CHD patients, the significant increase in the non-classical subpopulation of Asy and CHD is of great interest due to its restorative characteristics (**Figure 5**). This could also explain, together with the inhibition of pro-inflammatory mediators in the heart, the improvement in cardiac function that we have demonstrated in the murine model of mixed infection by *T. cruzi* under treatment with fenofibrate (Cevey et al., 2017; Rada et al., 2020). Further case control studies, namely, cohorts of fenofibrate vs. placebo treated patients, are needed to test the hypothesis that fenofibrate restores the percentage of CCR2^+^ non-classical monocytes leading to improvement of ventricular function in patients with Chagas disease.

In this work, the expression of HLA-DR in all the groups of patients was studied. We observed a trend to increase in HLA-DR expression in *T. cruzi* stimulated monocytes from CHD patients. Besides, fenofibrate tends to inhibit this effect (**Figure 6**). Furthermore, we have shown that HLA-DR tends to increase its expression in the classical monocyte subpopulation from CHD patients stimulated with *T. cruzi.* Fenofibrate treatment showed a tendency to restore HLA-DR expression to baseline levels (**Figure 7A**). However, another study shows that not only HLA-DR increased in the classical monocytes of cardiac patients but also in those of indeterminate. Likewise, in that work a lower expression of HLA-DR on intermediate monocytes of Asy patients was observed when compared to the CHD patients (Pinto et al., 2018). Deficiency in the expression of HLA-DR may reflect the severity of a disease. The low HLA-DR MFI constitutes an independent risk factor in predicting mortality, since decreased expression of HLA-DR on monocytes was associated with impaired antigen presentation and poor prognosis (Li et al., 2020). The fact that in our study fenofibrate treatment did not modify HLA-DR expression is encouraging, since it would mean that it does not inhibit antigen presentation capacity.

**Figure 8:**
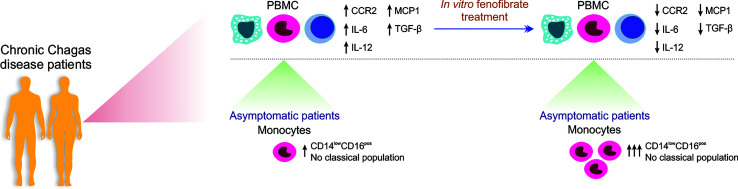
Schematic representation of the *in vitro* effect of Fenofibrate in PBMC and monocytes from patients with chronic Chagas disease. PBMC from Chagas disease patients display higher levels of IL-12, TGF-β, IL-6, MCP1, and CCR2 than cells from uninfected individuals. *In vitro* fenofibrate treatment exerts modulatory effect, decreasing the expression of CCR2, IL-6, IL-12, TGF-β, and MCP-1. Asymptomatic patients have a high percentage of non-classical monocytes, which increases even more after fenofibrate treatment.

In conclusion, monocyte profiling through analysis of inflammatory markers may be of value to direct appropriate coadjuvant therapies, like the one envisioned in this work by means of fenofibrate treatment, with the aim to help reduce the extensive and noxious inflammatory and profibrotic response arising in chronic Chagas heart disease patients (**Figure 8**).

## Data Availability Statement

The original contributions presented in the study are included in the article/**Supplementary Material**. Further inquiries can be directed to the corresponding author.

## Ethics Statement

The studies involving human participants were reviewed and approved by the Comité de Ética del Hospital General de Agudos Cosme Argerich, Ciudad Autónoma de Buenos Aires, Argentina. The patients/participants provided their written informed consent to participate in this study.

## Author Contributions

NG and AP designed experiments and analyzed data. NG, AP, GM, AC, and FP and contributed to the writing of the manuscript. AP, AC and FP did experiments. NP, AM, JG, and MG performed the clinical studies and the collection of blood samples. NG, GM, FP, AC, and AP contributed to final approval of the version to be published. All authors contributed to the article and approved the submitted version.

## Funding

This work was supported by the Universidad de Buenos Aires [Grant number 20020170100562BA] and the Agencia Nacional de Promoción Científica y Tecnológica [Grant number PICT 2016-0629].

## Conflict of Interest

The authors declare that the research was conducted in the absence of any commercial or financial relationships that could be construed as a potential conflict of interest.

## Publisher’s Note

All claims expressed in this article are solely those of the authors and do not necessarily represent those of their affiliated organizations, or those of the publisher, the editors and the reviewers. Any product that may be evaluated in this article, or claim that may be made by its manufacturer, is not guaranteed or endorsed by the publisher.

## Supplementary Material

Supplementary Figures 1 PBMC were treated *in vitro* or not with 100 µM of fenofibrate (Fen). After 48 h, IL-12, TGF-β, IL-6, MCP-1 and CCR2 mRNAs were measured in healthy individuals (HI) **(A)**. PBMC were treated or not with 100 µM of Fen. After 20 h according to CD14 and CD16 expression, they were classified as Classical (CD14high/CD16neg), Intermediate (CD14high/CD16pos) and Non-Classical (CD14low/CD16pos). The percentages of Classical, Intermediate and Non-Classical and are shown for HI, asymptomatic (Asy) and patients with Chagas heart disease (CHD) **(B)**. PBMC were treated or not with 100 µM of Fen. After 16-20 h, PBMC were classified according to CD14 and CCR2 expression. The percentages of CD14pos/CCR2pos monocytes are shown in HI, Asy and CHD, where each patient is represented by a circle **(C)**. PBMC were treated or not with 100 µM of Fen. The mean fluorescence intensity (MFI) of HLA-DR was determined in basal CD14pos cells after 20 h of Fen treatment. It shows the MFI of CD14pos/HLA-DRpos monocytes in HI, Asy and CHD patients, where each patient is represented by a circle **(D)**. For **(A)**, mRNA levels were determined by RT-qPCR and normalized against β-Actin. Results are expressed as the mean of 3 independent experiments. Differences between fenofibrate-treated PBMC were analyzed using the Wilcoxon test for paired samples and are shown as the mean of the experiments ± SEM. For **(B-D),** the data were analyzed by fitting a mixed effect model with a Tukey *post-hoc* test and the results are expressed as the mean of the experiments ± SEM.

Supplementary Figures 2 PBMC were treated or not with 100 µM of fenofibrate (Fen). After 20 h according to CD14 and CD16 expression, they were classified as Classical (CD14high/CD16neg), Intermediate (CD14high/CD16pos) and Non-Classical (CD14low/CD16pos) for healthy individuals (HI), asymptomatic (Asy) and patients with Chagas heart disease (CHD). It shows the percentage of classical, intermediate and non-classical monocytes with CCR2^+^ expression **(A)** and the mean fluorescence intensity (MFI) of classical, intermediate and non-classical monocytes with HLA-DR^+^ expression **(B).** For **(A, B)**, the results are shown as the mean of the experiments ± SEM. These data were analyzed by fitting a mixed effect model with a Tukey *post-hoc* test.
