# Supplementary figures and images for: Fenofibrate Increases the Population of Non-Classical Monocytes in Asymptomatic Chagas Disease Patients and Modulates Inflammatory Cytokines in PBMC
Source: Front Cell Infect Microbiol. 2022 Mar 11;11:785166. doi: 10.3389/fcimb.2021.785166 (PMC8963737; doi:10.3389/fcimb.2021.785166)

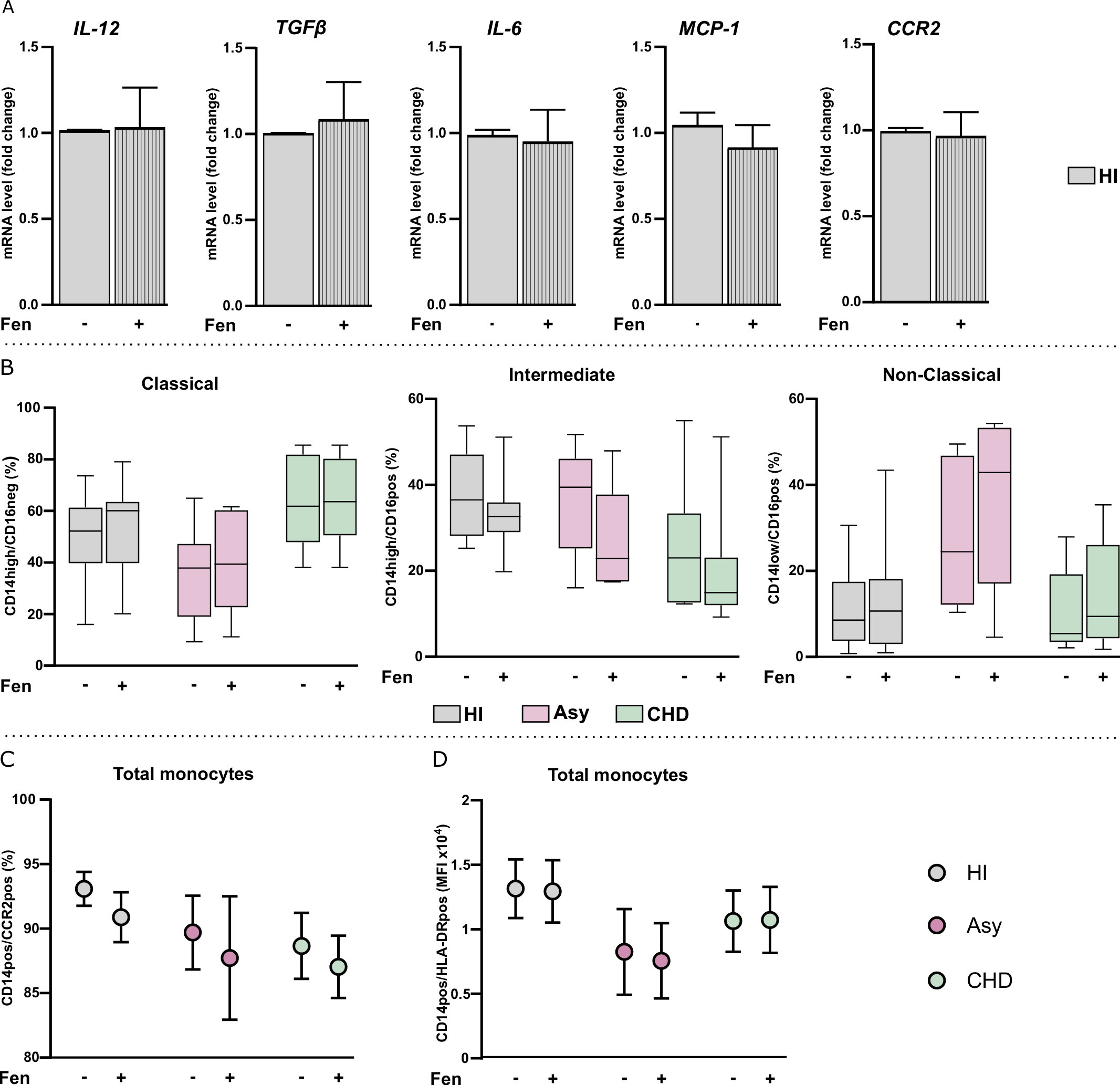

Supplement: Supplementary Figures 1 — PBMC were treated in vitro or not with 100 µM of fenofibrate (Fen). After 48 h, IL-12, TGF-β, IL-6, MCP-1 and CCR2 mRNAs were measured in healthy individuals (HI) (A). PBMC were treated or not with 100 µM of Fen. After 20 h according to CD14 and CD16 expression, they were classified as Classical (CD14high/CD16neg), Intermediate (CD14high/CD16pos) and Non-Classical (CD14low/CD16pos). The percentages of Classical, Intermediate and Non-Classical and are shown for HI, asymptomatic (Asy) and patients with Chagas heart disease (CHD) (B). PBMC were treated or not with 100 µM of Fen. After 16-20 h, PBMC were classified according to CD14 and CCR2 expression. The percentages of CD14pos/CCR2pos monocytes are shown in HI, Asy and CHD, where each patient is represented by a circle (C). PBMC were treated or not with 100 µM of Fen. The mean fluorescence intensity (MFI) of HLA-DR was determined in basal CD14pos cells after 20 h of Fen treatment. It shows the MFI of CD14pos/HLA-DRpos monocytes in HI, Asy and CHD patients, where each patient is represented by a circle (D). For (A), mRNA levels were determined by RT-qPCR and normalized against β-Actin. Results are expressed as the mean of 3 independent experiments. Differences between fenofibrate-treated PBMC were analyzed using the Wilcoxon test for paired samples and are shown as the mean of the experiments ± SEM. For (B-D), the data were analyzed by fitting a mixed effect model with a Tukey post-hoc test and the results are expressed as the mean of the experiments ± SEM. [file Image_1.tiff]

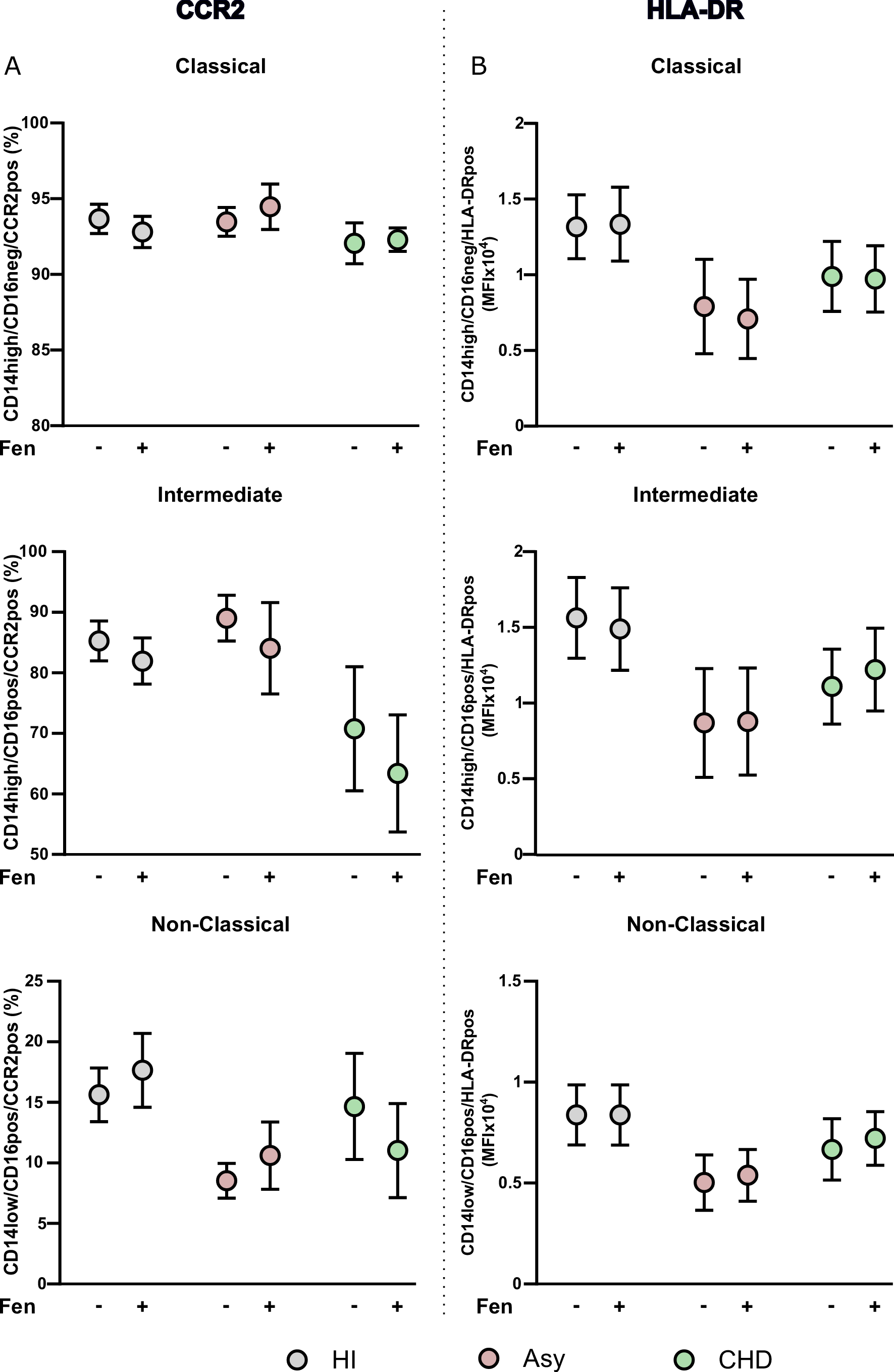

Supplement: Supplementary Figures 2 — PBMC were treated or not with 100 µM of fenofibrate (Fen). After 20 h according to CD14 and CD16 expression, they were classified as Classical (CD14high/CD16neg), Intermediate (CD14high/CD16pos) and Non-Classical (CD14low/CD16pos) for healthy individuals (HI), asymptomatic (Asy) and patients with Chagas heart disease (CHD). It shows the percentage of classical, intermediate and non-classical monocytes with CCR2+ expression (A) and the mean fluorescence intensity (MFI) of classical, intermediate and non-classical monocytes with HLA-DR+ expression (B). For (A, B), the results are shown as the mean of the experiments ± SEM. These data were analyzed by fitting a mixed effect model with a Tukey post-hoc test. [file Image_2.tiff]
